# A Novel Microwave-Induced Plasma Ionization Source for Ion Mobility Spectrometry

**DOI:** 10.1038/srep44051

**Published:** 2017-03-13

**Authors:** Jianxiong Dai, Zhongjun Zhao, Gaoling Liang, Yixiang Duan

**Affiliations:** 1 Analytical and Testing Center, Sichuan University, Chengdu 610064, P.R. China; 2 College of Chemical Engineering, Sichuan University, Chengdu 610065, P.R. China; 3 College of Chemistry, Sichuan University, Chengdu 610065, P.R. China; 4 Research Center of Analytical Instrumentation, Key Laboratory of Bio-resource and Eco-environment, Ministry of Education, College of Life Sciences, Sichuan University, Chengdu 610064, P.R. China

## Abstract

This work demonstrates the application of a novel microwave induced plasma ionization (MIPI) source to ion mobility spectrometry (IMS). The MIPI source, called Surfatron, is composed of a copper cavity and a hollow quartz discharge tube. The ion mobility spectrum of synthetics air has a main peak with reduced mobility of 2.14 cm^2^V^−1^s^−1^ for positive ion mode and 2.29 cm^2^V^−1^s^−1^ for negative ion mode. The relative standard deviations (RSD) are 0.7% and 1.2% for positive and negative ion mode, respectively. The total ion current measured was more than 3.5 nA, which is much higher than that of the conventional ^63^Ni source. This indicates that a better signal-to-noise ratio (SNR) can be acquired from the MIPI source. The SNR was 110 in the analysis of 500 pptv methyl tert-butyl ether (MTBE), resulting in the limit of detection (SNR = 3) of 14 pptv. The linear range covers close to 2.5 orders of magnitude in the detection of triethylamine with a concentration range from 500 pptv to 80 ppbv. Finally, this new MIPI-IMS was used to detect some volatile organic compounds, which demonstrated that the MIPI-IMS has great potential in monitoring pollutants in air.

Ion mobility spectrometry (IMS) is a molecular shape analyzer that can separate and detect gas-phase ions in ambient pressure, on the basis of their different velocities, under a relative weak electric field^1^. IMS was born under the name of plasma chromatography in the early 1970^2^. Since then, the applications of IMS were proposed in numerous fields because of low expenditure, fast response time (a few seconds), good portability, relatively low detection limits, etc. These applications include the detection of organic explosives^3^ and inorganic explosives^4^, chemical warfare agents^5^, illegal drugs^6^, food and feed analyses^7^, clinical analysis^8^,^9^, pharmaceutical applications^10^, environmental monitoring^11^, and process and bioprocess monitoring^12^.

In general, the IMS device was assembled with an ionization source, drift tube, signal processing system and sample introduction system. The ion source is one of the key components that determine the performance of IMS. Usually, a radioactive ^63^Ni foil with a half-life of about 100.1 years is constructed in the commercial instruments^13^. The radioactive ^63^Ni source has many advantages, such as high stability, long lifetime, low cost and no extra power supply requirements. However, the use of the radioactive ^63^Ni source was restricted by law, which limited its applications. Therefore, many researchers have been immersed in the development of nonradioactive ionization sources over the past few decades. So far, there were several nonradioactive ionization sources which have been successfully applied to IMS, including, laser ionization source^14^, photoionization source^15^,^16^, electrospray ionization source^17^, surface ionization source^18^, corona discharge ionization source^13^, glow discharge ionization source^19^, thermal ionization source^20^ and He plasma ionization source^21^.

In fact, some nonradioactive ionization sources suffer from the electrode oxidation and pollution (partial discharge). However, the MIPI source is free from electrode oxidation since the microwave induced plasma (MIP) can be generated in a hollow quartz tube. Moreover, the MIPI source has a longer lifetime and a higher sensitivity compared to the UV light (about 1 year)^21^. The MIP, working at GHz, has been described invented in the 1951^22^. After that, many different kinds of devices were developed for microwave induced plasma, such as the resonance chamber, Surfatron and the microwave plasma torch. In 1979, the Surfatron device was described by Hubert *et al*.^23^. Several years later, an improved version of Surfatron was reported by Selby and Hieftje^24^. From then on, the Surfatron device has been used in many studies, including as an excitation source for atomic emission spectrometry (AES)^25^, as absorption cell for atomic absorption spectroscopy (AAS)^26^, as well as an ionization source for inorganic mass spectrometer^27^,^28^. The MIPI source was also used as a desorption/ionization source at ambient pressure, highly sensitive organic mass spectrometry, which was initially developed in our research group^29^.

In this research, the MIPI source was first used as an ionization source for ion mobility spectrometry. Previously, a majority of ionization sources for IMS could be roughly separated into two major categories. In one case, the ion sources is belong to photoionization with no reactant ions^15^. Thus, sample molecules were directly ionized in the source region. In another case, the ion sources, such as the corona discharge source^13^,^30^ and the glow discharge source^31^ usually ionize sample molecules with reactant ions, and the process of ionization was mainly involved through a series of proton transfer reactions in positive ion mode or charge transfer in negative ion mode. Moreover, the reactant ion peak (RIP) can be used as a standard peak in practice^32^. As expected, the MIPI source also has the reactant ions in both positive and negative ion modes. When the sample gas was introduced, proton transfer reactions or charge transfer reactions were initiated between sample molecules and reactant ions in the ionization chamber. The product ions were pulsed into the drift region and forced by the electric strength through a shutter grid. These product ions arrived in order under a weak electric field on the faraday plate.

This study demonstrates a new ion source coupled with an IMS. The experiments were carried out to present the characteristics of the MIPI source (discharge gas flow rate and microwave power), the performance of MIPI-IMS (stability, limit of detection, and linear ranges) and the simple applications of the MIPI-IMS in the field of environmental monitoring. The comparison between the MIPI source and the traditional radioactive ^63^Ni source, or other nonradioactive ion sources, was also presented and discussed.

## Results and Discussion

In this study, initial characters of the MIPI-IMS were investigated. Selected peaks were used to evaluate the performance of MIPI-IMS. The reduced mobility (K_0_) of RIP in a weak electric field was calculated according to equation (1)^33^. The resolving power R was calculated using equation (2)^34^.


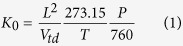



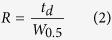


where L (cm) is the valid length of drift region, V (V) is the potential at shutter grid, T (K) is the drift gas temperature, P (Torr) is the drift gas pressure in the drift region, t_d_ (s) is the drift time for the ion through the drift region and W_0.5_ (s) is the peak width at half height.

The reduced mobility (K_0_) of product ion peak was calculated through standards. Many compounds including reactant ions were proposed as standards for the ion mobility spectrometry by Eiceman *et al*.^32^. So the K_0_ of product ions were calculated using equation (3)^35^.


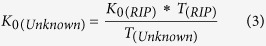


where *K*_0(*RIP*)_ is the reduced mobility of RIP, *K*_0(*Unkown*)_ is the reduced mobility of product ions, *T*_(*RIP*)_ is the drift time of the RIP, *T*_(*Unknown*)_ is the drift time of the product ions.

### Background mobility spectrum

It is very important to observe the background spectrum because it is closely related to the formation of product ions. The mobility spectrum and the mass spectrum in both positive and negative ion mode were presented.

#### Positive ion mode

**Figure 1:**
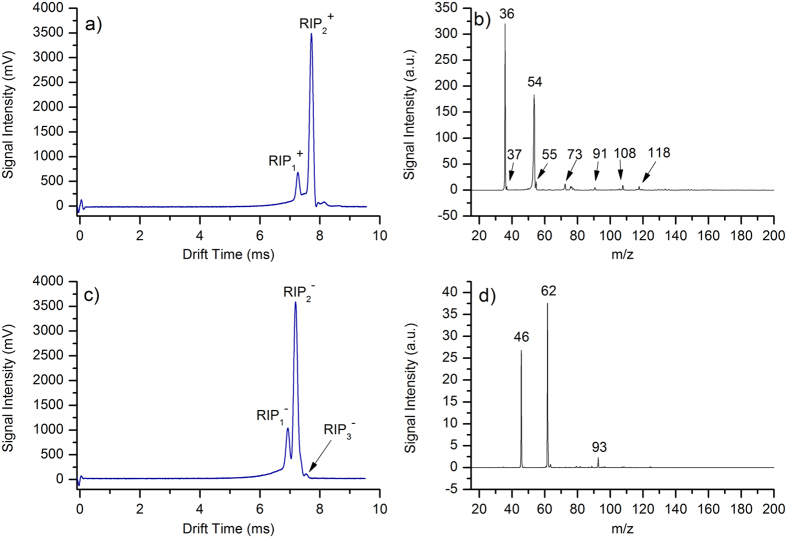
Background spectrum of air taken with MIPI-IMS in the (**a**) positive, (**c**) negative ion mode; Mass spectrum of background ions provided by the MIPI-IMS ionization chamber in the (**b**) positive, (**d**) negative ion mode.

Figure 1a shows the background of synthetic air taken with the unidirectional flow MIPI-IMS in positive ion mode. The two peaks are lower (RIP_1_^+^) at drift time of 7.27 ms with reduced mobility K_0_ 2.27 cm^2^V^−1^s^−1^ and a higher one (RIP_2_^+^) at drift time of 7.71 ms with reduced mobility K_0_ 2.14  cm^2^V^−1^s^−1^. In order to identify the chemical composition of these two peaks, an ion trap mass spectrometer (LCQ Fleet; Thermo Fisher Scientific, San Jose, CA) was used to analyze the ions produced by the MIPI-IMS ionization chamber. The mass range was set from m/z 15 to m/z 200 and a typical mass spectrum is shown in Fig. 1b. The major peak at m/z 36 was reported to be H_2_O·NH_4_^+^^36^. The other peaks are m/z 37 for (H_2_O)_2_H^+^, m/z 54 for (H_2_O)_2_NH_4_^+^, m/z 55 for (H_2_O)_3_H^+^, m/z 73 for (H_2_O)_4_H^+^, m/z 91 for (H_2_O)_5_H^+^, m/z 108 for (H_2_O)_5_ NH_4_^+^ and m/z 118 for (H_2_O)_4_N_2_NH_4_^+^^36^. These two peaks that appear in the mobility spectrum mainly consist of ammonia-based ions and hydronium-based ions in the positive ion mode. The parameters of the RIP_2_^+^ are as following: peak height, 3500 mV; ion current, 3.5 nA; peak width at half height; *W*_*0.5*_ = 0.136 ms; resolution, R = 56.7.

#### Negative ion mode

Figure 1c shows the ion mobility spectrum of a single run of synthetic air in negative ion mode. There are two weak peaks and a strong peak that appear in the mobility spectrum at 6.93 ms for RIP_1_^−^, 7.19 ms for RIP_2_^−^ and 7.54 ms for RIP_3_^−^, with reduced mobility K_0_ values of 2.38, 2.29 and 2.19 cm^2^V^−1^s^−1^, respectively. Figure 1d illustrates the mass spectrum with a mass range from m/z 15 to m/z 200. There are three major peaks at m/z 46 for NO_2_^−^, m/z 62 for NO_3_^−^ and m/z 93 for (HNO_2_)NO_2_^−^. Therefore, these three peaks that appear in the negative mobility spectrum mainly consisted of NO_2_^−^, NO_3_^−^ and (HNO_2_)NO_2_^−^, which was different from the O_2_^−^(H_2_O)_n_ obtained by the conventional ^63^Ni^11^. The parameters of the RIP_2_^−^ are as follows: peak height, 3500 mV; ion current, 3.5 nA; peak width at half height; *W*_*0.5*_ = 0.152 ms; resolution, R = 47.4.

The ion concentration for MIPI source is more than 2*10^10^ counts per second per cubic centimeter, which is higher than that obtained by ^63^Ni source. The experiment was detailed in supplementary. The stability of this new MIPI-IMS device was also investigated through monitoring the peak height of RIP_2_^+^ and RIP_2_^−^ every other minute in a period of 1 hour. The relative standard deviation (RSD) for the positive and negative ion mode was 0.7% and 1.2%, respectively. In fact, for more than two years, the signal intensity of RIP in both positive and negative ion modes were almost not attenuated during our experiments, which can be ascribed to the special discharge mode (nonpolar) of the MIPI source.

### Characteristics of the MIPI

The performance of the MIPI source was seriously affected by several operational parameters such as the microwave power, plasma gas flow rate, discharge gas species and the inner diameter of the hollow quartz discharge tube. The sensitivity of MIPI-IMS is closely related to the available reactant ions. Experiments showed that the larger the inner diameter of the hollow quartz tube helps to obtain more reactant ions without decreasing the resolution. However, the inner diameter was limited by the design and the outer diameter (6 mm). Due to the limitation in manufacturing, the inner diameter of 4 mm for the hollow quartz tube was selected. Both helium and argon gas can be used to produce microwave plasma. However, the ignition power of helium plasma (at least 100 W) is much higher than that of argon plasma (around 50 W)^29^. Additionally, the operational parameters of the microwave cavity need to be controlled strictly for the helium plasma^24^. Due to the reasons stated above, argon was chosen as discharge gas in this study.

**Figure 2:**
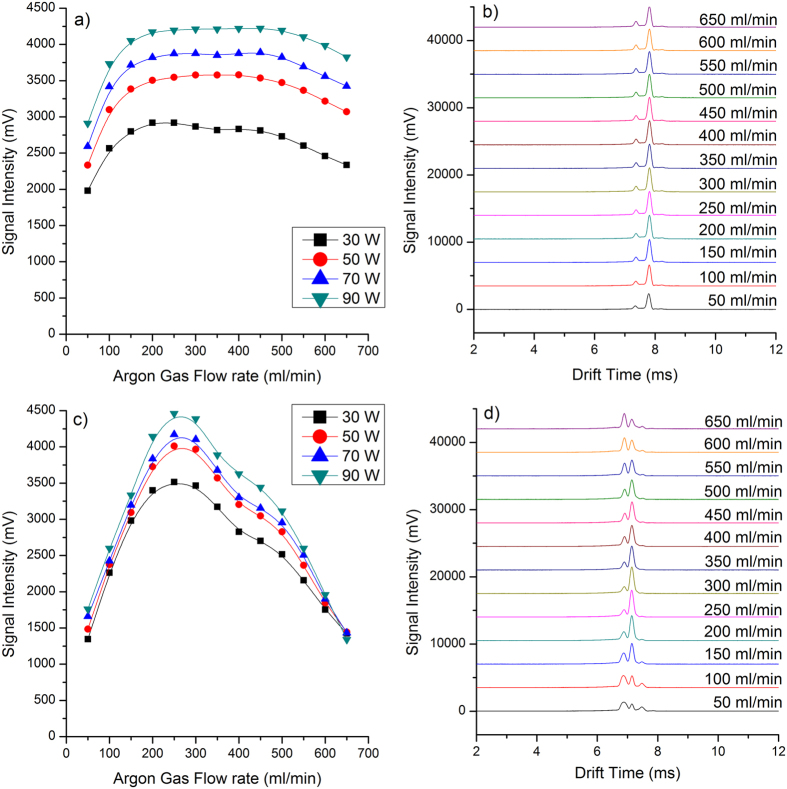
The peak height of (**a**) RIP_2_^+^; (**c**) RIP_2_^−^ as a function of argon gas flow rate under different microwave power. The background of air taken with MIPI-IMS with a microwave power of 50 W under different argon gas flow rates in (**b**) positive; (**d**) negative ion mode.

In addition, the influence of both microwave power and discharge (argon) gas flow rate on available reactant ions was detailed. Experiments showed that the microwave induced plasma became unstable when the argon gas flow rate was lower than 50 ml/min or higher than 650 ml/min. Therefore, the argon gas flow rate range was set from 50 to 650 ml/min. Figure 2a shows plots of signal intensity of RIP_2_^+^ versus the argon gas flow rate under different microwave powers in positive ion mode. As is shown in Fig. 2a, the peak height of RIP_2_^+^ raised rapidly with the increasing argon gas flow when the gas flow rate was lower than 200 ml/min. There were two aspects for this phenomenon. On one hand, the relatively higher argon gas flow rate increased the amount of plasma species, while on the other hand, more ions were swept into the source region from the hollow quartz tube by the relative higher gas flow rate. When the argon gas flow rate is higher than 200 ml/min, the signal intensity nearly keeps constant, which showed that the loss of ions and the generated ions reach a balance. When the argon gas flow rate is higher than 450 ml/min, the peak height of RIP decreased slowly with the increasing argon gas flow rate. It has to do with the dilution of the ions concentration rather than balance between production and losses. The influence of microwave power and argon gas flow rate on background of air was also investigated in negative ion mode and the results are shown in Fig. 2c. The peak height of RIP_2_^−^ increased exponentially when the argon gas flow rate is lower than 250 ml/min. When the argon gas flow rate is higher than 250 ml/min, the signal intensity of RIP_2_^−^ decreased rapidly. The variation trends of the peak height of RIP_2_^−^, in negative ion mode, were different from that of RIP_2_^+^ in positive ion mode.

**Figure 3:**
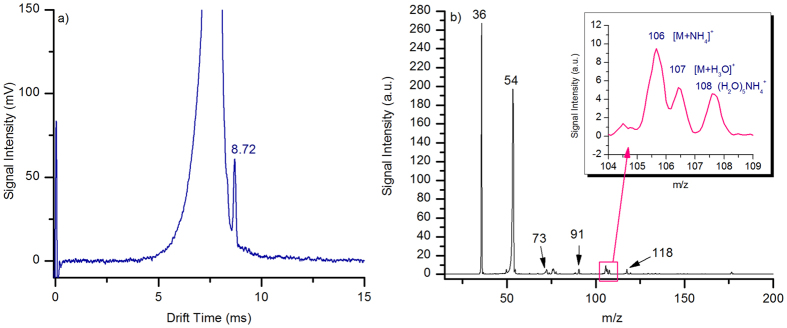
(**a**) Positive ion mobility spectrum of 500 pptv MTBE; (**b**) Mass spectrum for 500 pptv MTBE.

The mobility spectrum of synthetic air taken with MIPI-IMS with microwave power of 50 W under different argon gas flow rates in both positive and negative ion mode are shown in Figs 2b and 3d, respectively. The shape of peaks and the ratio of the peak’s heights of RIP_1_^+^ to RIP_2_^+^ almost remain the same with the increasing argon gas flow rate in positive ion mode. However, the shape and ratio of peaks were changed with different argon gas flow rates in negative ion mode. For example, the RIP_1_^−^ becomes fat when the argon gas flow is relatively low. The ratio of the peak height of RIP_1_^−^ to RIP_2_^−^ or RIP_3_^−^ to RIP_2_^−^ increased when decreasing or increasing the argon gas flow rate. In summary, the RIP_1_^−^ and RIP_3_^−^ gradually grow as the RIP_2_^−^ falls. This may be due to that the background ions in negative ion mode and were susceptible to the interference of discharge gas flow. Thus a change in the formation of background ions is the result. Although signal intensity of the reactant ion peaks increased with the increasing microwave power in both positive and negative ion mode, considering heat dispersion and power issues, the ignition power of 50 W was finally selected in our experiment. In fact, the sensitivity can be further improved by simply changing the microwave power in real world applications.

### Performance of MIPI-IMS

#### Detection of organic compound

Figure 3a shows the mobility spectrum of 500 pptv MTBE. Only one peak appeared in the mobility spectrum at drift time of 8.72 ms for 500 pptv MTBE. The peak width at half height *W*_*0.5*_ is 0.136 ms, so the resolving power is 64. The peak height is about 61 mV (SNR = 110) and the limits of detection is 14 pptv (SNR = 3). Therefore, the limits of detection for MIPI-IMS can reach the pptv level with a resolving power of 64. In fact, the gate pulse width can be changed, which could get a higher sensitivity or more resolving power for MIPI-IMS. The reduced mobility K_0_ is 1.89 cm^2^V^−1^s^−1^ calculated using equation 3. The reduced mobility for MTBE is lower than that obtained by UV-IMS with nitrogen as drift gas^37^, which can be ascribed to the formation of product ions. In order to identify the formation of product ions, the ions from the ionization chamber were introduced into the mass spectrometer. The mass spectrum with 500 pptv MTBE is shown in Fig. 3b. The peak at m/z 106 and 107 were confirmed as [M + NH_4_]^+^ and [M + H_3_O]^+^ by collision-induced dissociation (CID), which was detailed in supplementary. The major ions were [M + NH_4_]^+^, [M + H_3_O]^+^ and some rare [M + H]^+^ ions can be observed for MTBE at lower concentrations. For comparison, the product ion produced by UV-IMS was M^+^. Obviously, the size of the product ions produced by MIPI-IMS is larger than that obtained by UV-IMS. Therefore, the reduced mobility is lower than that obtained by UV-IMS.

#### Linear ranges

**Figure 4:**
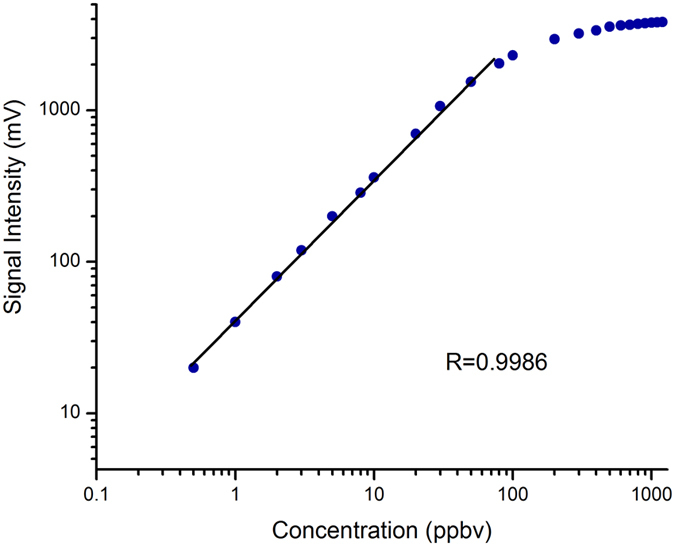
Signal intensity of triethylamine as a function of concentration.

The quantitative analysis capability of an IMS can be evaluated by a linear range. In order to obtain the linear dynamic ranges of the MIPI-IMS devices, triethylamine was used as a standard. Experiments showed that triethylamine created only one peak at different concentrations except for an ultra-high concentration (higher than 10 ppmv). Figure 4 shows the peak height versus the concentration of triethylamine with a concentration range of 0.5–1200 ppbv. The peak height rises linearly with the concentration of triethylamine from 0.5 to 80 ppbv, with the R at 0.9986. When the concentration of triethylamine exceeded 1200 ppbv, the RIP disappeared and the peak height of product ions was not further increased, even when the concentration of triethylamine was increased. Thus the linear dynamic range covers close to 2.5 orders of magnitude, which is little higher than that of the radioactive ^63^Ni IMS^32^. The wider linear dynamic range can be ascribed to the large amount of ions and chemical active species produced by the microwave plasma.

### Applications

#### Positive ion mode

**Figure 5:**
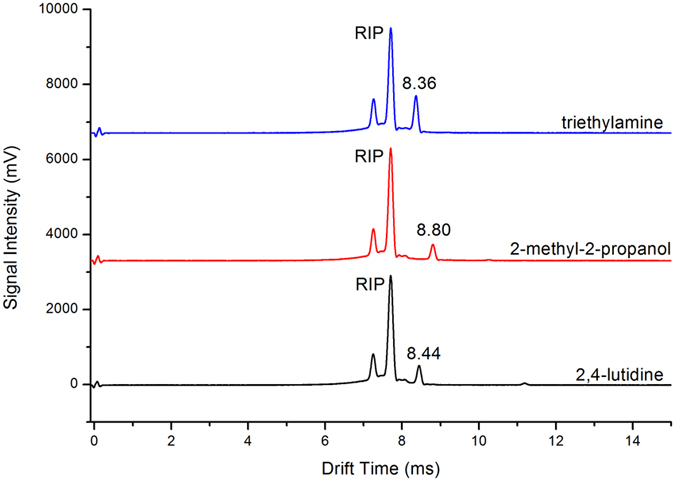
Positive ion mobility of triethylamine, 2-methyl-2-propanol and 2,4-lutidine.

Some volatile organic compounds such as triethylamine, 2-methyl-2-propanol and 2,4-lutidine were used to measure the qualitative analysis capability of this new MIPI-IMS in positive ion mode. The mobility spectra of these three volatile organic compounds are presented in Fig. 5. Each compound creates only one peak at a relatively lower concentration. An additional dimer ion peak for the increased concentration was observed for 2,4-lutidine and 2-methyl-2-propanol. These results are detailed in Table 1 with the reduced mobility K_0_ values to support the reported values. Experiments show that no fragment can be observed in the mobility spectrum except for an extremely high concentration (higher ppmv).

#### Negative ion mode

**Figure 6:**
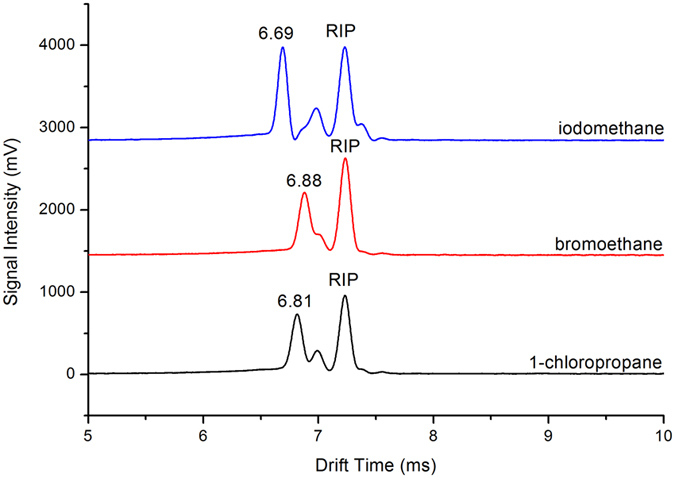
Negative ion mobility spectrum of iodomethane, bromoethane and 1-chloropropane, Conditions: gate pulse width 0.05 ms.

Three single halogenated compounds iodomethane, bromoethane and 1-chloropropane were analyzed using the negative ion mode of MIPI-IMS with a gate pulse width of 0.05 ms. Figure 6 shows the mobility spectrum for these three halogenated compounds. The results are summarized in Table 2. The product peaks for these three compounds results from the formation of (H_2_O)_n_X^−^ and (HNO_2_)_n_X^−^ (X = Cl, Br, I), which was identified by mass spectrum and the results were summarized in supplementary. The K_0_ values are lower than literature values, which can be ascribed to the formation of clusters with water molecule and HNO_2_. This can be explained by the MIPI-IMS being at room temperature, whereas the temperature in the literature was 130 °C.

## Conclusions

In this study, the performance of this new MIPI-IMS has been investigated. The experiments displayed that the MIPI-IMS can work in both positive and negative ion modes. The characteristics of the MIPI source in combination with IMS were investigated. The optimized discharge gas flow rate of 250 ml/min and the microwave power of 50 W were selected in the current design. In fact, the microwave power is flexible, and can be tuned for a higher sensitivity without reducing the resolving power in particular applications.

The MIPI-IMS can work in both positive and negative ion mode. The total current is higher than 3.5 nA with gate pulse width of 0.1 ms. The ion concentration is more than 2 ∗ 10^10^ counts per second per cubic centimeter, which is significantly higher than that of traditional radioactive ^63^Ni source. It means that the MIPI-IMS has a better signal to-noise ratio and detection capability. This new MIPI-IMS is a stable device in both the positive ion mode (RSD 0.7%) and the negative ion mode (RSD 1.2%).

Some selected volatile organic compounds were analyzed use MIPI-IMS and the product ions were mainly [M + H]^+^ or [M + NH_4_]^+^ verified by the mass spectrometer in positive ion mode. This new MIPI-IMS displayed excellent performance at high sensitivity (LOD 14 pptv) with a relatively high resolving power of 64. The quantitative analysis was also investigated and the results showed that the linear dynamic range covers close to 2.5 orders of magnitude, which is better than that of the traditional radioactive ^63^Ni IMS. Finally, some VOCs were analyzed by this new MIPI-IMS, which displayed that the MIPI-IMS has great potential in monitoring pollutants in the environment.

## Methods

### Sample preparation

HPLC grade 2,4-lutidine, triethylamine, MTBE, 2-methyl-2-propanol iodomethane, bromoethane and 1-chloropropane were purchased from Aladdin. The liquid samples were prepared by using exponential dilution method with cyclohexane as solvent. The gaseous samples were prepared via injecting 10 μl diluted liquid sample into a 0.5 L Tedlar bag filled with synthetic air. In order to ensure the completely evaporation of liquid samples, all the Tedlar bags containing sample were stored at room temperature for at least 120 minutes.

### Instrumentation

**Figure 7:**
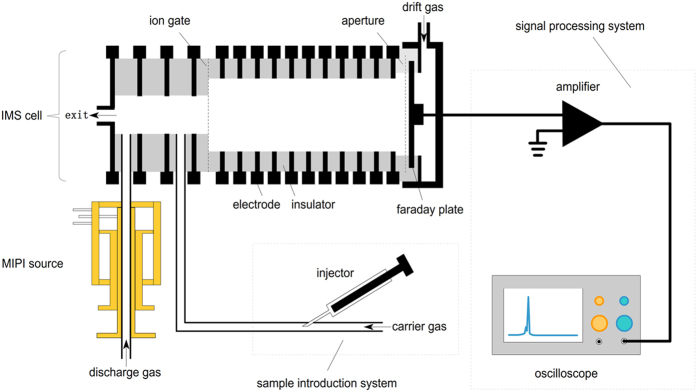
Schematic diagram of the MIPI-IMS experimental setup.

A schematic diagram of the MIPI-IMS is shown in Fig. 7. The MIPI-IMS device consists of four parts: the MIPI source, the IMS cell, the signal processing system and the sample introduction system. The homemade MIPI source was made of copper with a hollow quartz tube located in the center of the MIPI cavity axially. The microwave power was generated by a solid-state microwave generator (Nanjing Yanyou Electronic Science and Technology Co. Ltd. Nanjing, China), and transmitted to the MIPI cavity through a coaxial-cable. This generator can produce a power of 0–150 W with a frequency of 2.450 GHz. The forward and reflected microwave powers (V) can be read directly. The discharge gas such as argon or helium was introduced into the hollow quartz tube to absorb the microwave power. The plasma was ignited by a metal wire and then swept into the source region from the hollow quartz tube by the discharge gas. Specifically, the MIPI source was mounted perpendicular to the axis of drift tube in the experiments. In the axial arrangement, ions could be brought into the drift region even though the shutter grid was closed, because of the high flow velocity of the discharge gas. This may result in an unstable baseline.

The IMS cell consists of interlocked stainless steel guard rings separated by Teflon insulating rings with reaction region (4.8 cm long) and drift region (8.8 cm long). The drift region was constructed of stainless steel rings (i.d., 32 mm; 2 mm thick) and Teflon insulating rings (i.d., 32 mm; 6 mm thick). Each conduct rings was connected by a series of 1 MΩ 0.5 W resistors. A tunable high-voltage direct current (DC) power supply (8000 V, 2 mA) was used to form a homogeneous electric field. The shutter grid is a Bradbury–Nielsen type gate, which was used to retain the ions in the reaction region and inject pulsed ions into the drift region. This shutter grid was based on the printed circuit board (PCB) and the two sets of adjacent stainless steel wires (0.05 mm in diameter) were twined. The distance between the adjacent wires was 0.8 mm. A pulse generator provided two output voltages for the two sets adjacent wires. The rising and falling edges of the timing pulse were less than 5 μs. The shutter blocking voltage could be varied from 0 to 200 V. Typically the shutter blocking voltage used in these experiments is 50 V. The aperture grid was similar to the shutter grid. The aperture grid was grounded through a 390 kΩ resistor parallel with a 0.1 μF capacitor and placed in front of the collector with biased 1 mm spacing. The aperture grid was used to screen the potential induced by the ions and filter out pulse signals from the pulse generator. A current-to-voltage preamplifier with a gain of 10^9^ V/A was used to amplify the ion current. The output voltage was sent to a digital oscilloscope (Tektronix, MDO 3052) and then averaged multiple times. All measurements were obtained at room temperature with an acquisition frequency of 20 Hz.

A unidirectional flow design was used in the IMS instrument. The drift gas was introduced into the drift tube from the back of the collector to keep the drift tube free from contamination. The gaseous sample was injected into the sample introduction tube through a 500 μl injector (Hamilton 81265) regulated by an injection pump at a flow rate of 8 μl/min. The sample gas was diluted and swept into the ionization region by a carrier gas at a flow rate of 0.3 L/min. The discharge gases, drift gas and carrier gas, were provided by the Jin KeXing Gas Company. The discharge gases were high purity argon (99.999%) and high purity helium (99.999%). Both drift gas and carrier gas were synthetic air (80% N_2_, 20% O_2_) with 1–10 ppmv moisture. The experimental parameters in the experiments were summarized in Table 3 if not illustrated.

### Safety considerations

There are two points for safety. First, microwave radiation could be harmful for human beings. It is necessary to screen shield the microwave radiation using a metal mesh. It is also necessary to wear microwave protective clothing. Secondly, electrical shock may be happen when igniting the plasma with a slender metal wire. Thus insulating gloves should be worn.

## Additional Information

**How to cite this article**: Dai, J. *et al*. A Novel Microwave-Induced Plasma Ionization Source for Ion Mobility Spectrometry. *Sci. Rep.* **7**, 44051; doi: 10.1038/srep44051 (2017).

**Publisher's note:** Springer Nature remains neutral with regard to jurisdictional claims in published maps and institutional affiliations.

## Supplementary Material

Supplementary Material

## Figures and Tables

**Table 1:** The results of triethylamine, 2-methyl-2-propanol and 2,4-lutidine.

Compounds	Molecular weight	K_0_ in cm^2^V^−1^s^−1^	
		Measured	Literature
triethylamine	101	1.97	1.98^38^
2-methyl-2-propanol	74	1.87	1.83^39^
2,4-lutidine	107	1.95	1.95^13^

**Table 2:** The results for iodomethane, bromoethane and 1-chloropropane.

Compounds	Molecular weight	K_0_ in cm^2^ V^−1^ s^−1^	
		Measured	Literature
iodomethane	142	2.46	2.53^40^
bromoethane	109	2.40	2.63^40^
1-chloropropane	78.5	2.42	2.92^40^

**Table 3:** The instrumental parameters in experiments.

Operating parameters	setting
Voltage at ion gate	±4350 V
Gate pulse width	0.1 ms
Drift gas flow	1 L/min
Carrier gas flow	0.3 L/min
Discharge gas flow	0.25 L/min
Drift gas and carrier gas	Air
Discharge gas	Argon
Microwave power	50 W
Drift tube temperature	295 ± 2 K
Drift tube pressure	760 Torr
Gain of amplifier	10^9^ V/A
