# Supplementary material for: A Novel Microwave-Induced Plasma Ionization Source for Ion Mobility Spectrometry
Source: Sci Rep. 2017 Mar 13;7:44051. doi: 10.1038/srep44051 (PMC5347007; doi:10.1038/srep44051)
Supplement: Supplementary Material [file srep44051-s1.pdf]

Supplementary Material

A Novel Microwave-Induced Plasma Ionization Source for Ion Mobility Spectrometry

Jianxiong Dai[1], Zhongjun Zhao[3], Gaoling Liang[4], Yixiang Duan[2]*

1 Analytical and Testing Center, Sichuan University, Chengdu 610064, P.R. China

2 Research Center of Analytical Instrumentation, Key Laboratory of Bio-resource and Eco-environment, Ministry of Education, College of Life Sciences, Sichuan University, Chengdu 610065, P.R. China

3 College of Chemical Engineering, Sichuan University, Chengdu 610065, P.R. China

4 College of Chemistry, Sichuan University, Chengdu 610065, P.R. China

*Corresponding author: Prof. Yixiang Duan, Research Center of Analytical Instrumentation, Sichuan University, 29 Wangjiang Road, Chengdu 610064, China. E-mail: yduan@scu.edu.cn. Phone: (+86)028-85418180. Fax: (+86)028-85418180

Contents

**1. Ion concentration**
**Figure S-1** The experimental setup for measuring the ion current.
**2. Mass identification for MTBE**
**Figure S-2** The CID of a) m/z 106, b) m/z 107 for MTBE.
**3. Mass identification for halogenated compounds**
**Figure S-3** Mass spectra of a) 1-chloropropane, b) bromoethane, c) iodomethane.
**Figure S-4** The CID of Cl^- adductor
**Figure S-5** The CID of Br^- adductor
**Figure S-6** The CID of I^- adductor
**4. Reference**

1. Ion concentration

The ion current was measured by using source region of the IMS. Figure S-1 shows the experimental setup. Conditions: the pressure is 760 Torr, the temperature is 295 K, the drift gas was synthetic air with flow rate of 1 L/min, the voltage drop V = 3000 V, the discharge gas is argon with flow rate of 300 mL/min, the microwave power is 50 W, the inner diameter D = 16 mm, the length L = 45 mm.

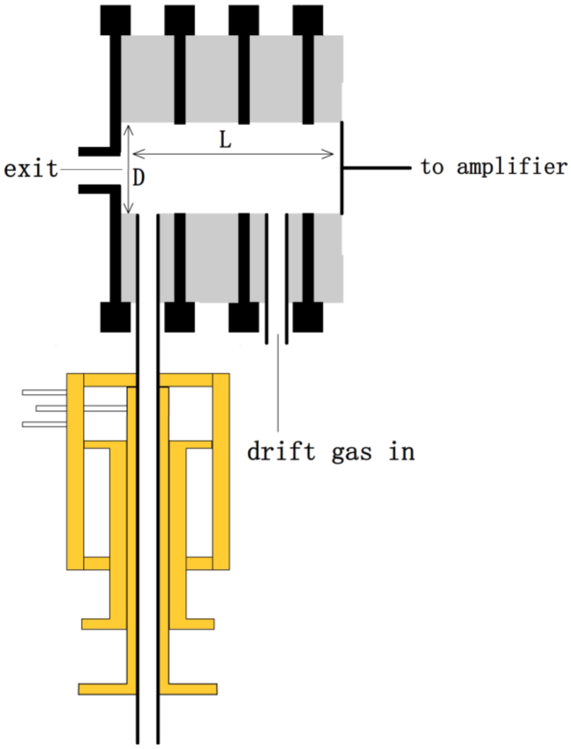

Figure S-1 The experimental setup for measuring the ion current.

The ion current was measured by a picoammeters, I = 30 nA for positive and negative ion mode. The number of ions n = Q/e = I*t/e = 30×10^-9*1/1.6×10^-19 = 1.88×10^11 counts*s^{-1}. The ion concentration c = n/(π*r^2*L) = 1.88×10^11/(3.14*0.8^2*4.5) = 2.08×10^10 counts*cm^{-3}*s^{-1}. Therefore, the measured ion concentration was more than 2*10^{10} counts per second per cubic centimeter, which is significantly higher than that obtained by ^{63}Ni source, 10^9 counts per second per cubic centimeter [1].

2. Mass identification for MTBE

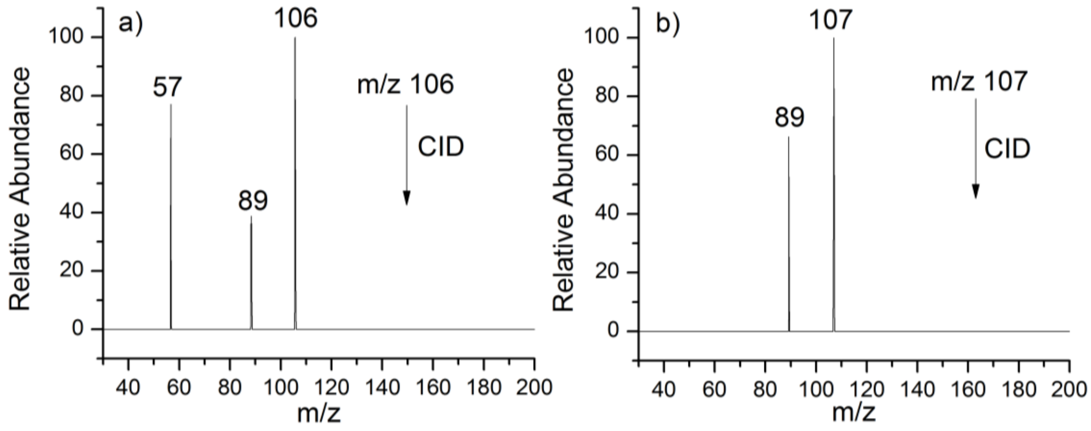

Figure S-2 The CID of a) m/z 106, b) m/z 107 for MTBE.

The collision-induced dissociation (CID) of m/z 106 and m/z 107 for MTBE is shown in Figure S-2. The peak at m/z 89 in Figure S-2a is the protonated molecule $[M+H]^+$, and the peak at m/z 57 is the fragment of MTBE, $(CH_3)_3C^+$. The peak at m/z 106 is $[M+NH_4]^+$, where the NH_4^+ is from the reactant ion of $H_2O\cdot NH_4^+$. The peak at m/z 107 was identified as $[M+H_3O]^+$ by CID from Figure S-2b.

3. Mass identification for halogenated compounds

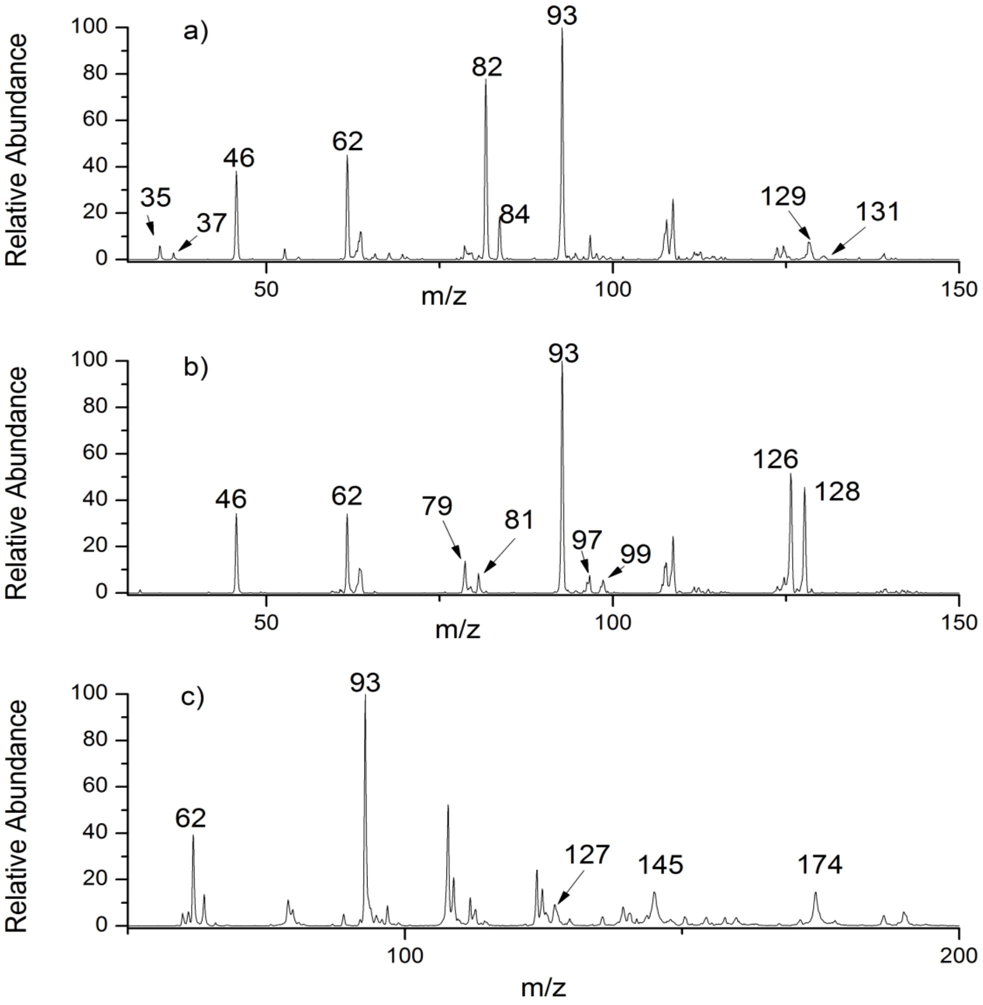

Figure S-3 Mass spectra of a) 1-chloropropane, b) bromoethane, c) iodomethane.

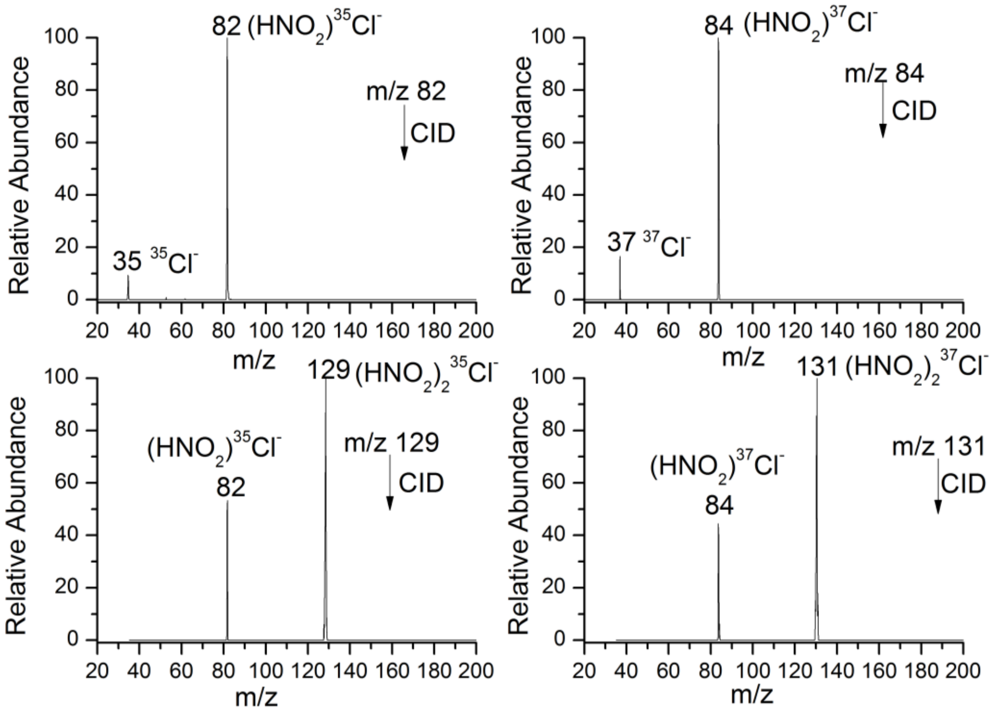

Figure S-4 The CID of Cl^- adductor

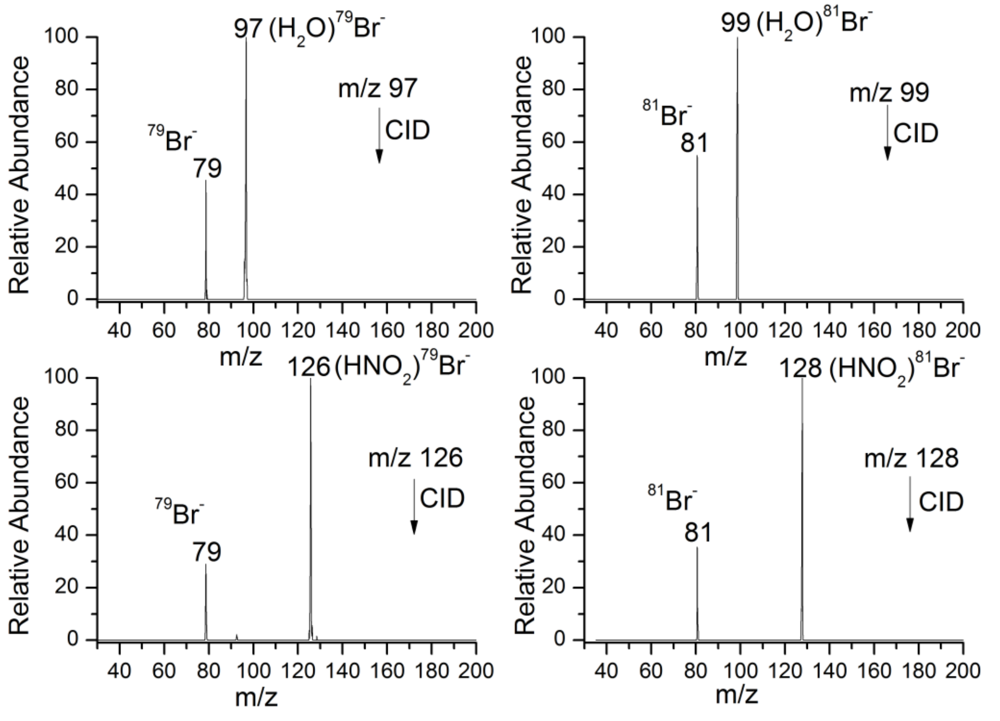

Figure S-5 The CID of Br^- adductor

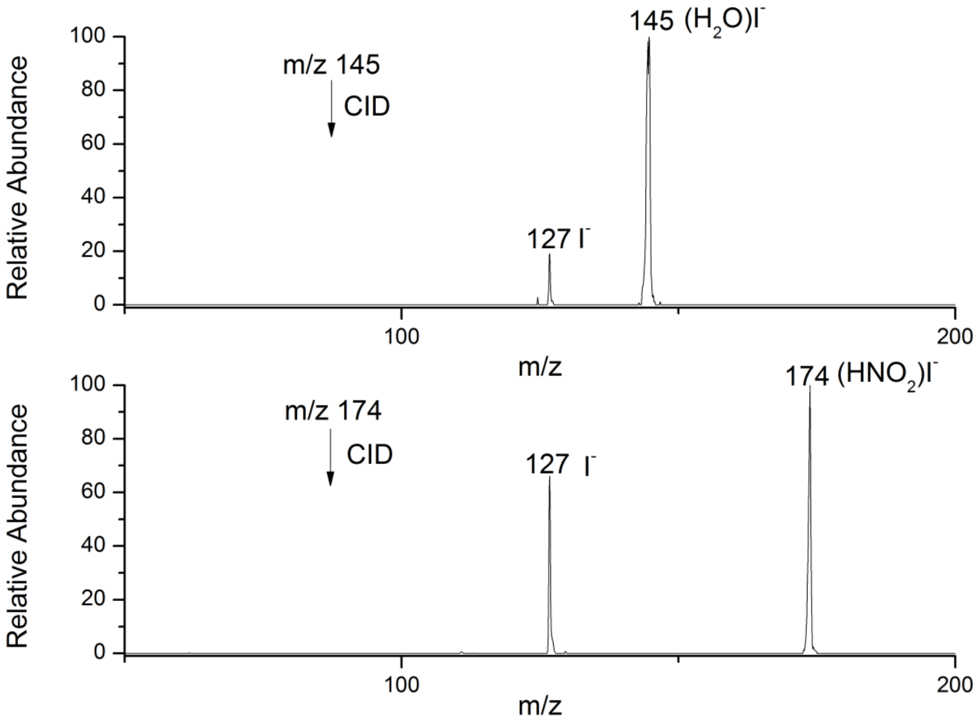

Figure S-6 The CID of I^- adductor

The mass spectra of the halogenated compounds are shown in Figure S-3. Figure S-3a shows the mass spectrum of 1-chloropropane. The peaks at m/z 35 and m/z 37 are chlorine isotope peaks. The peaks at m/z 82, m/z 84, m/z 129 and m/z 131 correspond to the adductor of Cl^-, which were confirmed by CID. As is shown in Figure S-4, the peaks at m/z 82, m/z 84, m/z 129 and m/z 131 were identified as $(HNO_2)^{35}Cl^-$, $(HNO_2)^{37}Cl^-$, $(HNO_2)_2{}^{35}Cl^-$ and $(HNO_2)_2{}^{37}Cl^-$.

The mass spectrum of bromoethane is shown in Figure S-3b. The peaks at m/z 79 and m/z 81 are the isotopic peaks of Br^-. The peaks at m/z 97, m/z 99, m/z 126 and m/z 128 were identified as $(H_2O)^{79}Br^-$, $(H_2O)^{81}Br^-$, $(HNO_2)\,^{79}Br^-$ and $(HNO_2)\,^{81}Br^-$. The CID for these peaks are given in Figure S-5.

The results for iodomethane are displayed in Figure S-3c. The peak at m/z 127 is I^-. The peaks at m/z 145 and m/z 174 are $(H_2O)\,I^-$ and $(HNO_2)\,I^-$, respectively. The CID for these two peaks are shown in Figure S-6.

4. Reference

1. Siegel, M.W., Atmospheric pressure ionization, in Plasma Chromatography, Carr, T.W., Ed., Plenum Press, New York, 1984, chap. 3, pp. 95-113
